# Adverse obstetric outcomes during delivery hospitalizations complicated by suicidal behavior among US pregnant women

**DOI:** 10.1371/journal.pone.0192943

**Published:** 2018-02-15

**Authors:** Qiu-Yue Zhong, Bizu Gelaye, Jordan W. Smoller, Paul Avillach, Tianxi Cai, Michelle A. Williams

**Affiliations:** 1 Department of Epidemiology, Harvard T.H. Chan School of Public Health, Boston, Massachusetts, United States of America; 2 Department of Psychiatry, Massachusetts General Hospital, Boston, Massachusetts, United States of America; 3 Psychiatric and Neurodevelopmental Genetics Unit, Center for Human Genetic Research, Massachusetts General Hospital, Boston, Massachusetts, United States of America; 4 Harvard Medical School, Boston, Massachusetts, United States of America; 5 Department of Biomedical Informatics, Harvard Medical School, Boston, Massachusetts, United States of America; 6 Children’s Hospital Informatics Program, Boston Children’s Hospital, Boston, Massachusetts, United States of America; 7 Department of Biostatistics, Harvard T.H. Chan School of Public Health, Boston, Massachusetts, United States of America; Universty of Edinburgh, UNITED KINGDOM

## Abstract

**Objective:**

The effects of suicidal behavior on obstetric outcomes remain dangerously unquantified. We sought to report on the risk of adverse obstetric outcomes for US women with suicidal behavior at the time of delivery.

**Methods:**

We performed a cross-sectional analysis of delivery hospitalizations from 2007–2012 National (Nationwide) Inpatient Sample. From the same hospitalization record, *International Classification of Diseases* codes were used to identify suicidal behavior and adverse obstetric outcomes. Adjusted odds ratios (aOR) and 95% confidence intervals (CI) were obtained using logistic regression.

**Results:**

Of the 23,507,597 delivery hospitalizations, 2,180 were complicated by suicidal behavior. Women with suicidal behavior were at a heightened risk for outcomes including antepartum hemorrhage (aOR = 2.34; 95% CI: 1.47–3.74), placental abruption (aOR = 2.07; 95% CI: 1.17–3.66), postpartum hemorrhage (aOR = 2.33; 95% CI: 1.61–3.37), premature delivery (aOR = 3.08; 95% CI: 2.43–3.90), stillbirth (aOR = 10.73; 95% CI: 7.41–15.56), poor fetal growth (aOR = 1.70; 95% CI: 1.10–2.62), and fetal anomalies (aOR = 3.72; 95% CI: 2.57–5.40). No significant association was observed for maternal suicidal behavior with cesarean delivery, induction of labor, premature rupture of membranes, excessive fetal growth, and fetal distress. The mean length of stay was longer for women with suicidal behavior.

**Conclusion:**

During delivery hospitalization, women with suicidal behavior are at increased risk for many adverse obstetric outcomes, highlighting the importance of screening for and providing appropriate clinical care for women with suicidal behavior during pregnancy.

## Introduction

Suicide is one of the leading causes of maternal mortality [1]. The strongest predictor of suicide is nonfatal suicidal thoughts and behaviors (hereafter referred to as suicidal behavior), which consists of suicidal ideation, plan, and attempt [2,3]. Pregnant women are more likely than the general population to endorse suicidal ideation, the thoughts of engaging in behaviors intended to end one’s life [2,4]. Depending on the timing of assessment, instruments used, and definitions adopted (Gentile 2011; Lindahl et al. 2005), the prevalence of suicidal ideation among pregnant women ranged from 5% to 33%. A rapid transition from the onset of ideation to the onset of planning and attempt has been shown in the literature [3,4]. Compared with suicidal ideation, suicide attempt, defined as engagement in potentially self-injurious behaviors in which there is at least some intent to die [2], is less common among pregnant women. The cumulative incidence of suicide attempt during pregnancy was 4 per 10,000 pregnancies in a large State of California database [5]. Despite decades of research, the causes and consequences of suicide and suicidal behavior during pregnancy remain uncertain [6]. To date, only six studies have assessed the association of suicidal behavior during pregnancy with maternal and neonatal outcomes [5, 7–11]. Only a limited range of outcomes and exposures has been evaluated and results have been inconsistent. For example, five of the six studies have focused primarily on suicide attempt by drug overdose during pregnancy and teratogenic effects [7–11]. Moreover, none of these six studies has evaluated the effect of suicidal ideation on pregnancy outcomes even though suicidal ideation is approximately 3.5 times more frequent than suicide attempt during pregnancy [12]. Due to sparse and inconsistent evidence on pregnancy outcomes, the effects of suicidal ideation or suicide attempt during pregnancy remain dangerously unquantified and under-studied.

Hence, the aim of our study was to assess the risk of adverse obstetric outcomes for women with suicidal behavior at the time of delivery. We hypothesized that suicidal behavior was associated with increased risk of adverse obstetric outcomes including cesarean delivery, longer length of stay, induction of labor, antepartum hemorrhage, placental abruption, postpartum hemorrhage, premature delivery, stillbirth, premature rupture of membranes, excessive fetal growth, poor fetal growth, fetal distress, and fetal anomalies. We used the National (Nationwide) Inpatient Sample (NIS), the largest publicly available all-payer inpatient health care database in the United States [13], to obtain a population-based sample of delivery hospitalizations during the period 2007–2012. We also evaluated the socio-demographic characteristics of the study population and characteristics of hospitals where women were hospitalized.

## Materials and methods

### Database

As a part of the Healthcare Cost and Utilization Project (HCUP), the NIS is the largest publicly available all-payer inpatient health care database in the United States sponsored by the Agency for Healthcare Research and Quality (AHRQ) [13]. The NIS approximates a stratified 20% sample of all US nonfederal, short-term, general, and other specificity hospitals, which contains discharge data on approximately 7 million hospital inpatient stays each year [14]. Five hospital characteristics—geographic region, ownership, location, teaching status, and bed size—are used for stratification [13]. Before 2012, within each stratum, a 20% stratified random sample of hospitals in the sampling frame was drawn by the AHRQ [13]. All discharges from sampled hospitals were included. Beginning with 2012, the NIS was redesigned to improve national estimates, and within each stratum, a 20% stratified random sample of discharges from all HCUP-participating hospitals was drawn by the AHRQ [15]. Discharge-level sampling weights based on the sampling scheme are available to obtain national estimates. The NIS does not allow linkage of patient’s discharge records. Since the data are publicly available and do not contain personal identifiers, this study is exempt from review by institutional review boards. This study conforms to the Data Use Agreement for the Nationwide Databases from the HCUP.

### Study population

This is a cross-sectional analysis of the of delivery hospitalizations, irrespective of the primary reasons for admission, from 2007–2012 National (Nationwide) Inpatient Sample. Similar to a previous study [16], our study population comprised women aged 12–55 years. The identification of delivery hospitalizations were predicated upon delivery-related *International Classification of Diseases*, Ninth Revision, Clinical Modification (ICD-9-CM) diagnosis and procedure codes and Diagnosis-Related Group (DRG) codes [16–18] (S1 Table).

### Variable specification

Exposures included in this analysis were suicidal ideation (thoughts of engaging in behaviors intended to end one’s life) [2] and suicide and self-inflicted injury (injuries in suicide, suicide attempt, and self-inflicted injuries specified as intentional) [19]. ICD-9-CM diagnosis codes were used to identify hospitalizations with any (primary or secondary) discharge diagnostic codes for suicidal ideation (V62.84) and suicide and self-inflicted injury (E950-959). The validity of these codes (E950-959) have been evaluated in previous studies [20,21]. For example, in a sample from a health maintenance organization in California, the positive predictive value for E950-959 was 86% [21]. Suicidal behavior-related hospitalizations were defined as hospitalizations with suicidal ideation or suicide and self-inflicted injury. Considering the relatively small number of hospitalizations for suicide and self-inflicted injury (*n* = 247), we did not present results for suicidal ideation and suicide and self-inflicted injury separately to be compliant with the HUCP Data Use Agreement.

Socio-demographic and baseline characteristics including age, race/ethnicity, median household income quartiles for patient zip code, expected primary payer, length of stay, and total charges were coded in the NIS. Median household income quartiles for patient zip code provided a quartile classification estimating income of residents based on the year of data collection [22]. Other baseline characteristics (ever smoking, previous cesarean delivery, non-psychotic depression, psychosis, and alcohol/substance abuse) were abstracted from the same delivery hospitalization record (primary or secondary discharge diagnostic codes) using ICD-9-CM diagnosis codes listed in S2 Table. Hospital characteristics were obtained directly from the data set, including hospital region, location, bed size, and teaching status.

Obstetric outcomes included in this analysis were: cesarean delivery, length of stay (in days) among cesarean and vaginal deliveries, induction of labor (applies to induction by cervical dilation and medical induction of labor), antepartum hemorrhage, placental abruption, postpartum hemorrhage, spontaneous delivery earlier than 37-week gestation, stillbirth, premature rupture of membranes, excessive fetal growth (applies to large-for-dates), poor fetal growth (applies to light-for-dates, placental insufficiency, and small-for-dates), fetal distress (applies to fetal metabolic acidemia), and fetal abnormality affecting management of mother (including conditions in the fetus that affecting management of mother: central nervous system malformation, chromosomal abnormality, hereditary disease in family possibly affecting fetus, suspected damage to fetus from viral disease/other disease in the mother, suspected damage to fetus from drugs or radiation, decreased fetal movements, and other known or suspected fetal abnormality, not elsewhere classified) (S2 Table).

### Statistical analyses

Using weights provided by the datasets, we reported national estimates representing discharges from all US community hospitals. We compared the distributions of socio-demographic, baseline, and hospital characteristics between women with and without suicidal behavior by performing Wald Chi-square and *t*-tests. We calculated odds ratios (ORs) and 95% confidence intervals (CIs) using logistic regression. To obtain adjusted odds ratios (aORs), we included maternal age (continuous), race/ethnicity, median household income quartiles for patient zip code, hospital region (Northeast, Midwest, South, West), hospital location (rural or rural), year, and smoking status as potential confounders in multivariable logistic regression models. All variables had <5% missing data except for race/ethnicity. We created missing indicator variables to address missing data for race/ethnicity and median household income quartiles for patient zip code. Total hospitalization charges were adjusted for inflation to reflect 2012 US dollars [23].

All analyses were conducted using SAS 9.4 (SAS Institute, Cary, NC, USA) and SAS-callable SUDAAN software (version 11.0.1, RTI International, Research Triangle, NC, USA). Statistical significance was set at two-sided *P*<0.05. Some computations were run on the Odyssey cluster supported by the Faculty of Arts & Sciences Division of Science, Research Computing Group at Harvard University.

### Sensitivity analyses

Given that multiple birth was associated with a myriad of complications such as preterm labor and fetal growth restriction [24], we further restricted our analyses to singletons. In addition to this method, we also adjusted for multiple birth in multivariable regression analyses. We also explored the associations of suicidal behavior with adverse outcomes stratified by the status of comorbid psychiatric disorders (including non-psychotic depression and substance/alcohol abuse).

## Results

After applying the NIS sampling weights, 23,507,597 delivery hospitalizations (unweighted: 4,915,185) were included in this study. Among these hospitalizations, 2,180 hospitalizations (unweighted: 456) had a diagnosis of suicidal ideation (*n =* 1,948) or suicide and self-inflicted injury (*n* = 247). The prevalence for suicidal behavior at delivery was 9.3 per 100,000 hospitalizations.

Table 1 shows the socio-demographic and baseline characteristics. The median age among women with and without suicidal behavior was 25.17 years and 26.88 years, respectively. Compared with women without suicidal behavior, women with suicidal behavior were more likely to be in the age group of 12–24 years (42.63% vs. 33.33%) and the lowest quartile of median household income for patient zip code (38.42% vs. 26.78%). Regarding race/ethnicity, Whites were the largest racial/ethnic group for women with and without suicidal behavior. The proportion of Blacks among women with suicidal behavior was higher (20.73% vs. 11.83%) as compared to women without suicidal behavior while the proportion of Whites was lower (35.53% vs. 44.08%). The majority of women with suicidal behavior (65.11%) had their medical care paid by Medicaid. Nearly half of women (49.96%) without suicidal behavior had private insurance as the expected primary payer while this proportion was 20.18% among women with suicidal behavior. In addition, women with suicidal behavior had longer length of hospital stays (4.47 days vs. 2.65 days) and larger total charges ($25,367 vs. $13,727 US dollars). Approximately 20.12% of women with suicidal behavior had diagnosis codes for ever smoking while only 5.22% of women without suicidal behavior had these diagnosis codes. The proportion of previous cesarean delivery was similar among women with (15.59%) and without (16.30%) suicidal behavior. The proportion of multiple gestations among women with and without suicidal behavior was 2.23% and 1.83%, respectively. Compared with women without suicidal behavior, women with suicidal behavior were more likely to have non-psychotic depression (49.05% vs. 1.97%), psychosis (37.06% vs. 0.70%), and substance/alcohol abuse (24.38% vs. 1.56%). Of note, 76.38% of women with suicidal behavior had comorbid psychiatric disorders (non-psychotic depression, psychosis, or substance/alcohol abuse), while only 3.87% of women without suicidal behavior had comorbid psychiatric disorders.

**Table 1:** Socio-demographic and baseline characteristics of women with and without suicidal behavior at delivery hospitalizations (N = 23,507,597).

Characteristics	Women				*P-*value
	With suicidal behavior (N = 2,180)		Without suicidal behavior (N = 23,505,417)		
	n	%	n	%	
**Age**, median (Q1, Q3), year	25.17 (21.14, 30.07)		26.88 (22.14, 31.54)		<0.0001
**Age categories**, year					
12–18	147	6.72	1,242,171	5.28	0.0025
19–24	783	35.91	6,593,376	28.05	
25–29	580	26.58	6,612,693	28.13	
30–34	458	21.01	5,627,271	23.94	
35–39	165	7.58	2,774,105	11.80	
40–55	48	2.19	655,800	2.79	
**Race/Ethnicity**					
White	775	35.53	10,361,272	44.08	<0.0001
Black	452	20.73	2,780,146	11.83	
Hispanic	394	18.06	4,560,404	19.40	
Asian or Pacific Islander	53	2.43	1,043,250	4.44	
Native American	44	2.02	177,870	0.76	
Other	170	7.80	983,174	4.18	
Missing	293	13.43	3,599,300	15.31	
**Median household income quartiles for patient zip code**					
Quartile 1 (poorest)	838	38.42	6,295,764	26.78	<0.0001
Quartile 2	550	25.22	5,802,547	24.69	
Quartile 3	431	19.76	5,681,732	24.17	
Quartile 4 (wealthiest)	265	12.17	5,272,670	22.43	
Missing	96	4.43	452,704	1.93	
**Expected primary payer**					
Medicare	109	5.01	163,430	0.70	<0.0001
Medicaid	1,420	65.11	10,115,279	43.03	
Private insurance	440	20.18	11,744,266	49.96	
Self-pay	113	5.19	738,774	3.14	
No charge	≤10^b^	N/A	49,282	0.21	
Other	93	4.28	652,330	2.78	
Missing	≤10^b^	N/A	42,056	0.18	
**Length of stay**, mean ± SE, day	4.47 ± 0.27		2.65 ± 0.01		<0.0001
**Total charges**^a^, mean ± SE, dollar	25,367 ± 2,607		13,727 ± 192		<0.0001
**Ever smoking**	439	20.12	1,226,010	5.22	<0.0001
**Previous cesarean delivery**	342	15.59	3,833,383	16.30	0.73

Individual cell counts may not add up to the global cell counts because of rounding and the differences arising from variance computations when using the discharge weights.

Percentages may not add up to 100% due to rounding, missing data, or data falling into categories too small to report.

Abbreviations: Q1, quartile 1; Q3, quartile 3

^a^ Total charges were adjusted for inflation to reflect 2012 US dollars.

^b^ HCUP privacy protection requirements do not allow the reporting of data where there are less than or equal to 10 individual records in a given cell.

Hospital characteristics are presented in Table 2. Pregnant women with suicidal behavior as compared with women without suicidal behavior were more likely to reside in the Northeast (20.56% vs. 16.16%) and Midwest region (24.66% vs. 21.36%) but not in the South region (30.36% vs. 37.97%). A larger proportion of women with suicidal behavior were admitted in a hospital with large bed size (71.53% vs. 62.41%) or a teaching hospital (69.07% vs. 46.76%).

**Table 2:** Characteristics of hospitals where women with and without suicidal behavior related-hospitalizations being hospitalized (N = 23,507,597).

Characteristics	Women				*P-*value
	With suicidal behavior (N = 2,180)		Without suicidal behavior (N = 23,505,417)		
	n	%	n	%	
**Region**					
Northeast	448	20.56	3,797,361	16.16	0.05
Midwest	538	24.66	5,021,140	21.36	
South	662	30.36	8,924,719	37.97	
West	533	24.43	5,762,198	24.51	
**Location**					
Rural	116	5.34	2,611,348	11.11	0.0007
Urban	2,036	93.36	20,686,094	88.01	
**Bed size**					
Small	126	5.76	2,521,780	10.73	0.0018
Medium	467	21.40	6,105,709	25.98	
Large	1,560	71.53	14,669,954	62.41	
**Teaching hospital**	1,506	69.07	10,991,857	46.76	<0.0001

Individual cell counts may not add up to the global cell counts because of rounding and the differences arising from variance computations when using the discharge weights.

Percentages may not add up to 100% due to rounding or missing data.

Table 3 shows the frequencies and ORs for obstetric outcomes among delivery hospitalizations. No significant association was observed for suicidal behavior with cesarean delivery (aOR = 0.95; 95% CI: 0.77–1.18) and induction of labor (aOR = 1.18; 95% CI: 0.94–1.48). Women with suicidal behavior were more likely to experience antepartum hemorrhage (aOR = 2.34; 95% CI: 1.47–3.74), placental abruption (aOR = 2.07; 95% CI: 1.17–3.66), postpartum hemorrhage (aOR = 2.33; 95% CI: 1.61–3.37), premature delivery (aOR = 3.08; 95% CI: 2.43–3.90), and fetal anomalies (aOR = 3.728; 95% CI: 2.57–5.40). An 11-fold increase odds for stillbirth (aOR = 10.73; 95% CI: 7.41–15.56) was found among offspring born to mothers with suicidal behavior. The association between suicidal behavior and premature rupture of membranes was not statistically significant (aOR = 1.41; 95% CI: 0.96–2.09). Compared with women without suicidal behavior, women with suicidal behavior had fetuses that were more likely to experience poor fetal growth (aOR = 1.70; 95% CI: 1.10–2.62) and less likely to experience excessive fetal growth (aOR = 0.49; 95% CI: 0.21–1.18). No significant association was found for maternal suicidal behavior with fetal distress (aOR = 1.13; 95% CI: 0.88–1.44). The mean length of stay was longer for women with suicidal behavior than for women without suicidal behavior (vaginal delivery: 3.43 days vs. 2.53 days; cesarean delivery: 6.35 days vs. 3.55 days).

**Table 3:** Obstetric outcomes among women with and without suicidal behavior during delivery hospitalizations (N = 23,507,597).

Obstetric outcomes	Women					OR (95% CI)	
	With suicidal behavior (N = 2,180)		Without suicidal behavior (N = 23,505,417)		Unadjusted	Adjusted^a^
	n	%	n	%		
**Cesarean delivery**	690	31.65	7,780,240	33.10	0.94 (0.76–1.16)	0.95 (0.77, 1.18)
**Length of stay**, mean ± SE, day						
** Vaginal delivery**	3.43 ± 0.57		2.53 ± 0.01		NA	NA
** Cesarean delivery**	6.35 ± 0.56		3.55 ± 0.02		NA	NA
**Induction of labor**	453	20.78	4,314,762	18.36	1.18 (0.94–1.47)	1.18 (0.94–1.48)
**Antepartum hemorrhage**	85	3.90	360,874	1.54	2.63 (1.64–4.21)	2.34 (1.47–3.74)
** Placental abruption**	56	2.57	249,838	1.06	2.47 (1.41–4.35)	2.07 (1.17–3.66)
**Postpartum hemorrhage**	140	6.42	659,363	2.81	2.39 (1.65–3.44)	2.33 (1.61–3.37)
**Spontaneous delivery <37-week gestation**	463	21.24	1,712,247	7.28	3.45 (2.74–4.33)	3.08 (2.43–3.90)
**Stillbirth**	171	7.84	154,685	0.66	12.84 (8.96–18.39)	10.73 (7.41–15.56)
**Premature rupture of membranes**	128	5.87	912,870	3.88	1.55 (1.06–2.26)	1.41 (0.96–2.09)
**Excessive fetal growth**	23	1.06	612,926	2.61	0.40 (0.17–0.97)	0.49 (0.21–1.18)
**Poor fetal growth**	101	4.63	513,837	2.19	2.17 (1.42–3.33)	1.70 (1.10–2.62)
**Fetal distress**	374	17.16	3,370,338	14.34	1.26 (0.98–1.61)	1.13 (0.88–1.44)
**Fetal anomalies**	126	5.78		342,232	1.46	4.15 (2.87–6.02)	3.72 (2.57–5.40)

Abbreviations: SE, standard error; OR, odds ratio; CI, confidence interval

^a^ Adjusted for maternal age (continuous), race/ethnicity, median household income quartiles for patient zip code, hospital region, hospital location, year, and smoking status

We explored the effect of multiple birth on the associations of suicidal behavior with adverse outcomes using two different methods (controlling for multiple birth or restricting to singletons). Multiple birth did not dramatically alter the reported effect estimates (S3 and S4 Tables). In separate sensitivity analyses, no material differences were seen in the ORs between suicidal ideation and suicide and self-inflicted injury (data not shown). For instance, the adjusted ORs of premature delivery for women with suicidal ideation only and suicide and self-inflicted injury irrespective of the presence or not of suicidal ideation were 3.45 (95% CI: 2.70–4.41) and 4.00 (95% CI: 2.06–7.77) among delivery hospitalizations. When stratified by the status of comorbid psychiatric disorders (S5 Table), the associations were generally stronger among women who did not have depression and alcohol/substance abuse, as compared with women who had depression or alcohol/substance abuse.

## Discussion

Our analyses revealed that hospitalized women with suicidal behavior at delivery were at significantly higher risk for a variety of adverse obstetric outcomes including antepartum hemorrhage, placental abruption, postpartum hemorrhage, and premature delivery. Strikingly, we observed a nearly 11-fold increased risk of stillbirth among offspring born to women with suicidal behavior at delivery. In addition, poor fetal growth and fetal anomalies were more likely to occur among the offspring of women hospitalized with suicidal behavior at delivery.

Several studies have assessed the adverse outcomes of suicidal behavior, more specifically, suicide attempt during pregnancy. Using the Vital Statistics-Patient Discharge database of California, Gandhi et al. [5] investigated maternal and neonatal outcomes among 2,132 pregnant women hospitalized for suicide attempt. Similar to our results, Gandhi et al. found that women who attempted suicide were not at statistically, significantly increased risk of premature rupture of membranes, primary cesarean delivery, and fetal distress. Contrary to our results, no significant association of suicide attempt with antepartum hemorrhage, placental abruption, or premature delivery was seen in the study by Gandhi et al. [5] One factor that might account for the discrepancy was that suicidal ideation was not included in the study by Gandhi et al. Another difference was that Gandhi et al. were able to link different hospitalizations for each woman while our analyses were strictly cross-sectional and we were not able to assesses multiple hospitalizations for each participant. A Danish population-based study [9] found no excess risk of adverse pregnancy outcomes including congenital abnormalities and preterm birth in 122 pregnant women who attempted suicide by drug overdose. An increased risk of miscarriage was reported in this population although the magnitude of risk estimates was not provided [9]. A series of studies conducted in Hungary has assessed the teratogenic effects of various drugs used by pregnant women who attempted suicide. The earliest population-based prospective study [8] concluded that drugs taken by 559 pregnant women who attempted suicide did not result in an increased risk for congenital abnormalities. Among the same study population, Czeizel et al. found an extremely high proportion of very early fetal loss after suicide attempt by self-poisoning in the first post-conceptual month [7]. A subsequent case-control study of 19 women who attempted suicide using very large doses of barbiturates during pregnancy and delivered live-born infants showed that the risk for congenital abnormalities or intrauterine fetal growth retardation was not increased when compared with 16 sibling controls who did not attempt suicide during pregnancy [11]. Finally, in their case-control study, Gidai et al., evaluated the teratogenic effect of nitrazepam on 43 pregnant women who attempted suicide and reported an OR of 3.8 (95% CI: 1.0–14.6) for congenital abnormalities compared with 29 sibling controls who did not attempt suicide [10]. Of note, inconsistent findings of suicide attempt with congenital abnormalities may be due, in part, to differences in the timing of suicide attempts, as the risks of structural congenital abnormalities will vary with the timing (e.g., the period of organogenesis). In addition, type and dose of drugs used by women attempting suicide may contribute to these inconsistent findings.

Due to the limited number of published studies on this topic, the mechanism of suicidal behavior with adverse obstetric outcomes has not yet been fully explored. As suggested by previous studies [7–11], drug teratogenicity could lead to adverse obstetric outcomes among women who attempted suicide by drug overdose during pregnancy. However, drug teratogenicity alone is not likely to explain the many statistically significant and moderate to strong associations observed in our study since the majority of our study population experienced suicidal ideation rather than suicide attempt. Therefore, we speculate that there are very likely other biological and psychosocial mechanisms that contribute to the observed associations between suicidal behavior and adverse obstetric outcomes. First, the clustering of adverse maternal characteristics, such as young age, low socioeconomic status, smoking, and obesity, may contribute to the increased risk of adverse outcomes among women with suicidal behavior. In the current study, we have taken into account some maternal characteristics, and most of the associations remained significant after adjusting for maternal age, race/ethnicity, and household income. We were unable to account for obesity given that the NIS database lacked information on the maternal body mass index (BMI). Second, comorbid disorders, especially comorbid psychiatric disorders, may also account for the increased risk of adverse obstetric outcomes. The association of psychiatric disorders with suicidal behavior is well-established [25]. In our study population, more than three quarters (76.38%) of women with suicidal behavior had comorbid psychiatric disorders. Maternal psychiatric disorders have been shown to be associated with a number of adverse obstetric outcomes [26–28]. Several potential causal pathways through which maternal psychiatric disorders may lead to adverse obstetric outcomes have been proposed, including hyperactivity of the hypothalamic-pituitary-adrenal axis [29]. Although the results were inconsistent, some statistically significant associations between psychotropic medication use during pregnancy and adverse obstetric outcomes have been identified [30–35]. Psychiatric disorders may also be indirectly associated with adverse obstetric outcomes through poor health behaviors during pregnancy, such as poor diet, poor weight gain, poor sleep, alcohol, tobacco, or caffeine use, and use of over the-counter medication [30]. Third, it is also possible that suicidal behavior may share some common underlying pathophysiological mechanisms with adverse obstetric outcomes. The stress-diathesis model suggests that suicidal behavior is the result of an interaction between state-dependent (environmental) stressors and a trait-like diathesis or susceptibility to suicidal behavior, independent of psychiatric disorders [36]. Genetic effects, childhood abuse, and epigenetic mechanisms, which may be involved in the etiology of the diathesis to suicidal behavior [37], could also play a role in the pathogenesis of adverse obstetric outcomes. To understand the mechanisms by which suicidal behavior increases the risk of certain adverse obstetric outcomes, future prospective studies are necessary that take into account antecedents of suicidal behavior, the methods of suicide attempt, timing of suicidal behavior during pregnancy, underlying socioeconomic status, comorbid diseases, especially comorbid psychiatric disorders, treatment effect of comorbid diseases, and previous history of psychiatric disorders and obstetric complications.

Consistent with previous studies [5,12], we found that pregnant women with suicidal behavior were younger; they were more likely to be Blacks, with lower socioeconomic status, and insured by Medicare or Medicaid. These characteristics may inform future prevention programs geared to identifying women at risk [38]. Intervention programs addressing socio-economic inequality by focusing on income-level interventions that move under-resourced pregnant women out of poverty [39] have been associated with improved mental health outcomes. In addition, efforts are warranted to develop tailored, culturally informed prevention programs for black pregnant women [12].

Strengths of this study included the population based design and very large sample size, facilitating the study of the rare event (i.e., suicidal behavior) during delivery hospitalization and the applicability to all U.S. pregnant women. Some of the limitations of the present study are directly related to the use of an administrative, inpatient database, such as the absence of information pertaining to maternal use of medication, the incompleteness of diagnosis codes, and the cross-sectional nature of the design. The accuracy and completeness of ICD-9-CM codes included in the NIS database were limited, leading to potential misclassifications of exposures and outcomes. Of note, the extent of misclassifications might be different between suicidal ideation and suicide and self-inflicted injury in this inpatient sample, given that suicidal ideation in itself may be unlikely to lead to hospitalization. One study showed that suicidal ideation was less likely to be coded than suicide attempt [40]. Further research is needed to evaluate the validity of these diagnosis codes, especially for suicidal ideation (V62.84) in obstetric population. The cross-sectional design limited our ability to assess causal and temporal relationships. Some adverse obstetric outcomes might contribute to an increased risk of suicidal behavior. For example, we observed a more than 11-fold increased risk of stillbirth, which could serve as a trigger for suicidal behavior. Future population-based, longitudinal studies are needed to explore the temporal relation between suicidal behavior during pregnancy and the onset of adverse obstetric outcomes. In addition, we did not separate suicide and self-inflicted injury from suicidal ideation due to the small number of suicide and self-inflicted injury in this database. Our sensitivity analysis found no material difference in the ORs between suicidal ideation and suicide and self-inflicted injury, highlighting the importance of studying suicidal ideation during pregnancy. Besides being a significant predictor of suicide attempt [2], suicidal ideation by itself may increase the risk for various adverse outcomes. Furthermore, although we adjusted for several potential confounders, we cannot exclude the possibility of some residual confounding from unmeasured covariates such as maternal BMI. Residual confounding, resulting from misclassification of covariates for which we adjusted, cannot be completely excluded.

## Conclusions

Within the context of these limitations, this study represents one of the first large population-based studies that describes a broad range of obstetric outcomes of women hospitalized with suicidal behavior during delivery. Although the prevalence of suicidal behavior during delivery is low, the association of suicidal behavior (irrespective of the types of suicidal behavior) with a series of adverse obstetric outcomes highlights the importance of screening for and providing appropriate clinical care for women with suicidal behavior in pregnancy. Clinicians and pregnant women need to be educated about the adverse outcomes of suicidal behavior in pregnancy. The burden of adverse perinatal outcomes may be attenuated if future prevention is undertaken by identifying women at high-risk for suicidal behavior, including women with lower socioeconomic status and those who are from ethnic minorities by providing them appropriate psychosocial treatment [5], income-level interventions, and culturally informed prevention programs [12]. Given the inherent limitations of hospital discharge data, future population-based, longitudinal studies with detailed data are needed on the methods of suicide attempt, timing of suicidal behavior, comorbid diseases and the treatment, and previous history of psychiatric disorders and obstetric complications to better understand the mechanisms by which suicidal behavior during pregnancy increases the risk of adverse obstetric outcomes.

## Supporting information

S1 Table International classification of diseases, Ninth Revision, Clinical Modification (ICD-9-CM) diagnosis and procedure codes, Diagnosis-Related Group (DRG) codes used to determine delivery-related hospitalizations. (DOCX)

S2 Table International Classification of Diseases, Ninth Revision, Clinical Modification (ICD-9-CM) diagnosis and procedure codes used to determine selected baseline characteristics and obstetric outcomes. (DOCX)

S3 Table Obstetric outcomes among women with and without suicidal behavior during delivery hospitalizations (N = 23,507,597). (DOCX)

S4 Table Obstetric outcomes among women with and without suicidal behavior during singleton delivery hospitalizations (N = 23,076,251). (DOCX)

S5 Table Obstetric outcomes among women with and without suicidal behavior according to the status of depression and alcohol/substance abuse during delivery hospitalizations (N = 23,507,597). (DOCX)
