# Supplementary material for: Adverse obstetric outcomes during delivery hospitalizations complicated by suicidal behavior among US pregnant women
Source: PLoS One. 2018 Feb 15;13(2):e0192943. doi: 10.1371/journal.pone.0192943 (PMC5814027; doi:10.1371/journal.pone.0192943)
Supplement: S1 Table — (DOCX) [file pone.0192943.s001.docx]

**S1 Table. International Classification of Diseases, Ninth Revision, Clinical Modification (ICD-9-CM) diagnosis and procedure codes, Diagnosis-Related Group (DRG) codes used to determine delivery-related hospitalizations**

| **Inclusion criteria** | |
| --- | --- |
| ICD-9-CM Diagnosis codes: | |
| V27 | Outcome of delivery |
| 650 | Normal Delivery |
| ICD-9-CM Procedure codes: | |
| 72 | Forceps, vacuum, and breech delivery |
| 73.22 | Internal and combined version with extraction |
| 73.59 | Other manually assisted delivery |
| 73.6 | Episiotomy |
| 74.0 | Classical cesarean section |
| 74.1 | Low cervical cesarean section |
| 74.2 | Extra peritoneal cesarean section |
| 74.4 | Cesarean section of other specified type |
| 74.99 | Other cesarean section of unspecified type |
| DRG codes: | |
| 370 | Cesarean section with complications, comorbidities |
| 371 | Cesarean section without complications, comorbidities |
| 372 | Vaginal delivery with complicating diagnoses |
| 373 | Vaginal delivery without complicating diagnoses |
| 374 | Vaginal delivery with sterilization&/or dilation & curettage |
| 375 | Vaginal delivery with operating room procedure except sterilization &/or dilation& curettage |
| **Exclusion criteria** | |
| ICD-9-CM Diagnosis codes: | |
| 630-639 | Ectopic and molar pregnancy and other pregnancy with abortive outcome |
| ICD-9-CM Procedure codes: | |
| 69.01 | Dilation and curettage for termination of pregnancy |
| 69.51 | Aspiration curettage of uterus for termination of pregnancy |
| 74.91 | Hysterectomy to terminate pregnancy |
| 75.0 | Intra-amniotic injection for abortion |
