# Supplementary material for: Adverse obstetric outcomes during delivery hospitalizations complicated by suicidal behavior among US pregnant women
Source: PLoS One. 2018 Feb 15;13(2):e0192943. doi: 10.1371/journal.pone.0192943 (PMC5814027; doi:10.1371/journal.pone.0192943)
Supplement: S2 Table — (DOCX) [file pone.0192943.s002.docx]

**S2 Table. International Classification of Diseases, Ninth Revision, Clinical Modification (ICD-9-CM) diagnosis and procedure codes used to determine selected baseline characteristics and obstetric outcomes**

| **ICD-9-CM Diagnosis codes** |  |
| --- | --- |
| Ever smoking | 305.1, V15.82, 649.0 |
| Previous cesarean delivery | 654.2 |
| Multiple birth | V27.2-V27.7, 651 |
| Non-psychotic depression | 296.2, 296.3, 296.82, 300.4, 301.12, 309.0, 309.1, 309.28, 311 |
| Psychosis | 295, 296, 297, 298 |
| Alcohol/substance abuse | 291, 292, 303, 304, 305, 648.3, 655.5, 965.0, V65.42 |
| Vaginal delivery | 72.0-72.4, 72.7 |
| Cesarean delivery | 699.7 |
| Antepartum hemorrhage | 641.1,641.2, 641.3, 641.8, 641.9 |
| Placental abruption | 641.2 |
| Postpartum hemorrhage | 666.0, 666.1, 666.2, 666.3 |
| Spontaneous delivery < 37-week gestation | 644.2 |
| Stillbirth | 656.4, V27.1, V27.3, V27.4, V27.6, V27.7 |
| Premature rupture of membranes | 658.10, 658.11, 658.13 |
| Excessive fetal growth | 656.6 |
| Poor fetal growth | 656.5 |
| Fetal distress | 656.3 |
| Fetal abnormality affecting management of mother | 655 |
| **ICD-9-CM Procedure codes:** |  |
| Cesarean delivery | 74.0, 74.1, 74.2, 74.4, 74.99 |
| Induction of labor | 73.1, 73.4 |
