# Supplementary material for: Adverse obstetric outcomes during delivery hospitalizations complicated by suicidal behavior among US pregnant women
Source: PLoS One. 2018 Feb 15;13(2):e0192943. doi: 10.1371/journal.pone.0192943 (PMC5814027; doi:10.1371/journal.pone.0192943)
Supplement: S3 Table — (DOCX) [file pone.0192943.s003.docx]

**S3 Table. Obstetric outcomes among women with and without suicidal behavior during delivery hospitalizations (N = 23,507,597)**

| **Obstetric outcomes** | **Women** | | | | |  | **OR (95% CI)** | |  |
| --- | --- | --- | --- | --- | --- | --- | --- | --- | --- |
|  | **With suicidal behavior**  **(N = 2,180)** | |  | **Without suicidal behavior**  **(N = 23,505,417)** | |  | **Unadjusted** | **Adjusted^a^** | **Adjusted^b^** |
|  | **n** | **%** |  | **n** | **%** |  |  |  |  |
| **Cesarean delivery** | 690 | 31.65 |  | 7,780,240 | 33.10 |  | 0.94 (0.76 - 1.16) | 0.97 (0.79 - 1.21) | 0.96 (0.78 - 1.19) |
| **Length of stay**, mean ± SE, day |  |  |  |  |  |  |  |  |  |
| **Vaginal delivery** | 3.43 ± 0.57 | |  | 2.53 ± 0.01 | |  | NA | NA | NA |
| **Cesarean delivery** | 6.35 ± 0.56 | |  | 3.55 ± 0.02 | |  | NA | NA | NA |
| **Induction of labor** | 453 | 20.78 |  | 4,314,762 | 18.36 |  | 1.18 (0.94 - 1.47) | 1.18 (0.94 - 1.48) | 1.18 (0.94 - 1.49) |
| **Antepartum hemorrhage** | 85 | 3.90 |  | 360,874 | 1.54 |  | 2.63 (1.64 - 4.21) | 2.62 (1.64 - 4.19) | 2.59 (1.61 - 4.15) |
| **Placental abruption** | 56 | 2.57 |  | 249,838 | 1.06 |  | 2.47 (1.41 - 4.35) | 2.39 (1.36 - 4.19) | 2.36 (1.34 - 4.16) |
| **Postpartum hemorrhage** | 140 | 6.42 |  | 659,363 | 2.81 |  | 2.39 (1.65 - 3.44) | 2.32 (1.61 - 3.36) | 2.30 (1.59 - 3.32) |
| **Spontaneous delivery <37-week gestation** | 463 | 21.24 |  | 1,712,247 | 7.28 |  | 3.45 (2.74 - 4.33) | 3.30 (2.61 - 4.17) | 3.38 (2.67 - 4.29) |
| **Stillbirth** | 171 | 7.84 |  | 154,685 | 0.66 |  | 12.84 (8.96 - 18.39) | 11.38 (7.87 - 16.44) | 11.49 (7.90 - 16.70) |
| **Premature rupture of membranes** | 128 | 5.87 |  | 912,870 | 3.88 |  | 1.55 (1.06 - 2.26) | 1.46 (0.99 - 2.16) | 1.45 (0.98 - 2.15) |
| **Excessive fetal growth** | 23 | 1.06 |  | 612,926 | 2.61 |  | 0.40 (0.17 - 0.97) | 0.46 (0.19 - 1.10) | 0.46 (0.19 - 1.11) |
| **Poor fetal growth** | 101 | 4.63 |  | 513,837 | 2.19 |  | 2.17 (1.42 - 3.33) | 2.00 (1.31 - 3.08) | 1.99 (1.29 - 3.07) |
| **Fetal distress** | 374 | 17.16 |  | 3,370,338 | 14.34 |  | 1.26 (0.98 - 1.61) | 1.16 (0.90 - 1.48) | 1.16 (0.91 - 1.48) |
| **Fetal abnormalities** | 126 | 5.78 |  | 342,232 | 1.46 |  | 4.15 (2.87 - 6.02) | 3.98 (2.74 - 5.78) | 3.97 (2.73 - 5.76) |

Abbreviations: SE, standard error; OR, odds ratio; CI, confidence interval

^a^ Adjusted for maternal age (continuous), race, median household income quartiles for patient zip code, hospital location, hospital region, and year

^b^ Further adjusted for multiple birth
