# Supplementary material for: Adverse obstetric outcomes during delivery hospitalizations complicated by suicidal behavior among US pregnant women
Source: PLoS One. 2018 Feb 15;13(2):e0192943. doi: 10.1371/journal.pone.0192943 (PMC5814027; doi:10.1371/journal.pone.0192943)
Supplement: S4 Table — (DOCX) [file pone.0192943.s004.docx]

**S4 Table. Obstetric outcomes among women with and without suicidal behavior during singleton delivery hospitalizations (N = 23,076,251)**

| **Obstetric outcomes** | **Women** | | | | |  | **OR (95% CI)** | |
| --- | --- | --- | --- | --- | --- | --- | --- | --- |
|  | **With suicidal behavior**  **(N = 2,132)** | |  | **Without suicidal behavior**  **(N = 23,074,119)** | |  | **Unadjusted** | **Adjusted^a^** |
|  | **n** | **%** |  | **n** | **%** |  |  |  |
| **Cesarean delivery** | 657 | 30.82 |  | 7,453,937 | 32.30 |  | 0.93 (0.75 - 1.16) | 0.96 (0.77 - 1.20) |
| **Length of stay**, mean ± SE, day |  |  |  |  |  |  |  |  |
| **Vaginal delivery** | 3.43 ± 0.57 | |  | 2.52 ± 0.01 | |  | NA | NA |
| **Cesarean delivery** | 6.23 ± 0.58 | |  | 3.46 ± 0.02 | |  | NA | NA |
| **Induction of labor** | 453 | 21.25 |  | 4,280,089 | 18.55 |  | 1.20 (0.96 - 1.50) | 1.20 (0.95 - 1.51) |
| **Antepartum hemorrhage** | 82 | 3.85 |  | 347,070 | 1.50 |  | **2.63 (1.63 - 4.25)** | **2.61 (1.62 - 4.21)** |
| **Placental abruption** | 53 | 2.49 |  | 240,327 | 1.04 |  | **2.41 (1.34 - 4.33)** | **2.32 (1.29 - 4.15)** |
| **Postpartum hemorrhage** | 131 | 6.14 |  | 633,300 | 2.74 |  | **2.32 (1.59 - 3.39)** | **2.26 (1.54 - 3.30)** |
| **Spontaneous delivery <37-week gestation** | 425 | 19.93 |  | 1,514,097 | 6.56 |  | **3.56 (2.80 - 4.53)** | **3.36 (2.64 - 4.29)** |
| **Stillbirth** | 162 | 7.60 |  | 138,982 | 0.60 |  | **13.56 (9.39 - 19.58)** | **11.78 (8.06 - 17.20)** |
| **Premature rupture of membranes** | 128 | 6.00 |  | 872,440 | 3.78 |  | **1.64 (1.12 - 2.39)** | **1.54 (1.04 - 2.27)** |
| **Excessive fetal growth** | 23 | 1.08 |  | 612,074 | 2.65 |  | 0.40 (0.17 - 0.97) | 0.46 (0.19 - 1.11) |
| **Poor fetal growth** | 96 | 4.50 |  | 477,119 | 2.07 |  | **2.23 (1.45 - 3.45)** | **2.04 (1.32 - 3.16)** |
| **Fetal distress** | 374 | 17.54 |  | 3,327,225 | 14.42 |  | 1.26 (0.99 - 1.62) | 1.18 (0.92 - 1.51) |
| **Fetal abnormalities** | 126 | 5.91 |  | 329,881 | 1.43 |  | **4.33 (2.99 - 6.29)** | **4.16 (2.86 - 6.04)** |

Abbreviations: SE, standard error; OR, odds ratio; CI, confidence interval

^a^ Adjusted for maternal age (continuous), race, median household income quartiles for patient zip code, hospital location, hospital region, and year
