# Supplementary material for: Adverse obstetric outcomes during delivery hospitalizations complicated by suicidal behavior among US pregnant women
Source: PLoS One. 2018 Feb 15;13(2):e0192943. doi: 10.1371/journal.pone.0192943 (PMC5814027; doi:10.1371/journal.pone.0192943)
Supplement: S5 Table — (DOCX) [file pone.0192943.s005.docx]

**S5 Table. Obstetric outcomes among women with and without suicidal behavior according to the status of depression and alcohol/substance abuse during delivery hospitalizations (N=23,507,597)**

| **Obstetric outcomes** | **Women** | |  | **OR (95% CI)** | |
| --- | --- | --- | --- | --- | --- |
|  | **With suicidal behavior**  **(N = 2,180)** | **Without suicidal behavior**  **(N = 23,505,417)** |  | **Unadjusted** | **Adjusted**^a^ |
|  |  |  |  |  |  |
| **Depression or alcohol/substance abuse** | 1,356 | 794,782 |  |  |  |
| **Cesarean delivery** | 478 | 282,716 |  | 0.99 (0.77, 1.27) | 1.01 (0.78, 1.30) |
| **Length of stay**, mean (SE), day |  |  |  |  |  |
| **Vaginal delivery** | 4.78 (0.36) | 2.79 (0.03) |  | NA | NA |
| **Cesarean delivery** | 6.38 (0.66) | 4.05 (0.04) |  | NA | NA |
| **Induction of labor** | 300 | 144,206 |  | 1.28 (0.98, 1.68) | **1.35 (1.03, 1.76)** |
| **Antepartum hemorrhage** | 57 | 24,707 |  | 1.37 (0.76, 2.45) | 1.31 (0.73, 2.36) |
| **Placental abruption** | 32 | 19,005 |  | 1.00 (0.47, 2.12) | 0.94 (0.44, 2.00) |
| **Postpartum hemorrhage** | 83 | 27,784 |  | 1.80 (1.10, 2.95) | **1.73 (1.05, 2.85)** |
| **Spontaneous delivery <37-week gestation** | 295 | 100,844 |  | 1.91 (1.43, 2.56) | **1.81 (1.34, 2.43)** |
| **Stillbirth** | 104 | 9,854 |  | 6.61 (4.29, 10.19) | **5.89 (3.76, 9.25)** |
| **Premature rupture of membranes** | 90 | 41,068 |  | 1.31 (0.82, 2.09) | 1.21 (0.75, 1.97) |
| **Excessive fetal growth** | 19 | 16,654 |  | 0.65 (0.24, 1.73) | 0.77 (0.29, 2.03) |
| **Poor fetal growth** | 58 | 31,917 |  | 1.07 (0.60, 1.91) | 1.09 (0.61, 1.94) |
| **Fetal distress** | 255 | 135,295 |  | 1.13 (0.85, 1.51) | 1.08 (0.81, 1.45) |
| **Fetal abnormalities** | 83 | 22,291 |  | 2.27 (1.40, 3.68) | **2.20 (1.35, 3.57)** |
| **No depression and alcohol/substance abuse** | 824 | 22,710,635 |  |  |  |
| **Cesarean delivery** | 213 | 7,497,524 |  | 0.72 (0.49, 1.06) | 0.77 (0.52, 1.13) |
| **Length of stay**, mean (SE), day |  |  |  |  |  |
| **Vaginal delivery** | 2.50 (0.41) | 2.53 (0.01) |  | NA | NA |
| **Cesarean delivery** | 6.29 (1.09) | 3.54 (0.02) |  | NA | NA |
| **Induction of labor** | 153 | 4,170,556 |  | 1.00 (0.70, 1.43) | 1.02 (0.72, 1.46) |
| **Antepartum hemorrhage** | 28 | 336,167 |  | 2.37 (1.05, 5.36) | **2.45 (1.09, 5.50)** |
| **Placental abruption** | 24 | 230,834 |  | 2.96 (1.24, 7.09) | **2.88 (1.18, 7.01)** |
| **Postpartum hemorrhage** | 57 | 631,579 |  | 2.61 (1.51, 4.54) | **2.51 (1.44, 4.38)** |
| **Spontaneous delivery <37-week gestation** | 168 | 1,611,403 |  | 3.37 (2.27, 5.01) | **3.31 (2.21, 4.95)** |
| **Stillbirth** | 67 | 144,830 |  | 13.92 (8.02, 24.14) | **12.66 (7.22, 22.19)** |
| **Premature rupture of membranes** | 37 | 871,802 |  | 1.21 (0.62, 2.35) | 1.17 (0.59, 2.31) |
| **Excessive fetal growth** | ≤10 ^b^ | 596,272 |  | 0.22 (0.03, 1.51) | 0.26 (0.04, 1.76) |
| **Poor fetal growth** | 43 | 481,920 |  | 2.55 (1.34, 4.85) | **2.34 (1.23, 4.46)** |
| **Fetal distress** | 119 | 3,235,043 |  | 1.00 (0.65, 1.54) | 0.95 (0.62, 1.44) |
| **Fetal abnormalities** | 43 | 319,941 |  | 3.87 (2.03, 7.40) | **3.70 (1.91, 7.15)** |

Abbreviations: OR, odds ratio; CI, confidence interval

^a^ Adjusted for maternal age (continuous), race/ethnicity, median household income quartiles for patient zip code, hospital region, hospital location, and year

^b^ HCUP privacy protection requirements do not allow the reporting of data where there are less than or equal to 10 individual records in a given cell
